# Supplementary material for: Spatiotemporal prediction of wildfire size extremes with Bayesian finite sample maxima
Source: Ecol Appl. 2019 Jun 20;29(6):e01898. doi: 10.1002/eap.1898 (PMC6851762; doi:10.1002/eap.1898)
Supplement: Supplementary file 1 [file EAP-29-na-s001.pdf]

# Spatiotemporal prediction of wildfire size extremes with Bayesian finite sample maxima

## Appendix S1: Prior specifications

Maxwell B. Joseph*[1], Matthew W. Rossi[1], Nathan P. Mietkiewicz[1], Adam L. Mahood[1], Megan E. Cattau[1], Lise Ann St. Denis[1], R. Chelsea Nagy[1], Virginia Iglesias[1], John T. Abatzoglou[2], and Jennifer K. Balch[1]

[1] *Earth Lab, University of Colorado Boulder*

[2] *Department of Geography, University of Idaho*

Prior distributions were chosen to regularize coefficients on the distribution specific means $\beta^{(\mu)}$ and structural zero parameters $\beta^{(\pi)}$. We used a regularized horseshoe prior on these coefficients, which shrinks irrelevant coefficients towards zero, while regularizing nonzero coefficients (Piironen, Vehtari, and others 2017). For zero-inflated models, we used a multivariate version of the regularized horseshoe (Peltola et al. 2014):

$$\begin{pmatrix} \beta_j^{(\mu)} \\ \beta_j^{(\pi)} \end{pmatrix} \sim \mathrm{N}\left( \mathbf{0}, \begin{pmatrix} \tau_1^2 \tilde{\lambda}_{1,j}^2 & \rho \tau_1 \tau_2 \tilde{\lambda}_{1,j} \tilde{\lambda}_{2,j} \\ \rho \tau_1 \tau_2 \tilde{\lambda}_{1,j} \tilde{\lambda}_{2,j} & \tau_2^2 \tilde{\lambda}_{2,j}^2 \end{pmatrix} \right),$$

$$\tilde{\lambda}_{m,j}^2 = \frac{c_m^2 \lambda_j^2}{c_m^2 + \tau_m^2 \lambda_j^2},$$

for each response dimension $m = 1, 2$ and coefficient $j = 1, ..., p$. Here $\rho$ is a correlation parameter, $\tau_1$ and $\tau_2$ are global variance hyperparameters, $c_1$ and $c_2$ are hyperparameters that determine the amount of shrinkage on the largest coefficients, and $\lambda_j$ is a local scale parameter drawn from a half-Cauchy distribution that control the amount of shrinkage applied to coefficient $j$ (Piironen, Vehtari, and others 2017). With this prior specification, information can be shared across the two response dimensions through the correlation pa-

*maxwell.b.joseph@colorado.edu

rameter $\rho$, and/or through the local scale parameters $\lambda_j$. For count models without structural zeros (the Poisson and negative binomial models), this multivariate prior simplifies to a univariate regularized horseshoe prior.

Spatiotemporal random effects were constructed using a temporally autoregressive, spatially intrinsically autoregressive formulation (Besag and Kooperberg 1995; Banerjee, Carlin, and Gelfand 2014). Temporarily suppressing the superscript that indicates whether the effects are on $\mu$ or $\pi$, and denoting column $t$ from an $S \times T$ $\mathbf{\Phi}$ as $\boldsymbol{\phi}_t$ we have:

$$\boldsymbol{\phi}_{t=1} \sim \mathrm{N}(\mathbf{0}, (\tau^{(\phi)}(\mathbf{D} - \mathbf{W}))^{-1})$$

$$\boldsymbol{\phi}_t \sim \mathrm{N}(\eta\boldsymbol{\phi}_{t-1}, (\tau^{(\phi)}(\mathbf{D} - \mathbf{W}))^{-1}), \quad t = 2, ..., T$$

where $\eta$ is a temporal dependence parameter, $\tau^{(\phi)}$ is a precision parameter, $\mathbf{D}$ is an $S \times S$ diagonal matrix with entries corresponding to the number of spatial neighbors for each spatial unit, and $\mathbf{W}$ is an $S \times S$ spatial adjacency matrix with nonzero elements only when spatial unit $i$ is a neighbor of spatial unit $j$ ($w_{i,j} = 1$ if $i$ is a neighbor of $j$, and $w_{i,j} = 0$ otherwise, including $w_{i,i} = 0$ for all $i$). $\tau^{(\phi)}$ is a precision parameter. We imposed a soft identifiability constraint that places high prior mass near $\sum_{s=1}^{S} \phi^*_{t,s} = 0$ for all $t$.

We applied a univariate regularized horseshoe prior to all $\beta$ coefficients in burned area models (Piironen, Vehtari, and others 2017):

$$\beta_j \sim \mathrm{N}\left(0, \tau^2\tilde{\lambda}_j^2\right), \quad \tilde{\lambda}_j^2 = \frac{c^2\lambda_j^2}{c^2 + \tau^2\lambda_j^2},$$

Spatiotemporal random effects were constructed in the same way as for the count models.

# Literature cited


Banerjee, Sudipto, Bradley P Carlin, and Alan E Gelfand. 2014. *Hierarchical Modeling and Analysis for Spatial Data.* CRC Press.

Besag, Julian, and Charles Kooperberg. 1995. "On Conditional and Intrinsic Autoregressions." *Biometrika* 82 (4). Oxford University Press: 733–46.

Peltola, Tomi, Aki S Havulinna, Veikko Salomaa, and Aki Vehtari. 2014. "Hierarchical Bayesian Survival Analysis and Projective Covariate Selection in Cardiovascular Event Risk Prediction." In *Proceedings of the Eleventh Uai Conference on Bayesian Modeling Applications Workshop-Volume 1218*, 79–88. CEUR-WS. org.

Piironen, Juho, Aki Vehtari, and others. 2017. "Sparsity Information and Regularization in the Horseshoe and Other Shrinkage Priors." *Electronic Journal of Statistics* 11 (2). The Institute of Mathematical Statistics; the Bernoulli Society: 5018–51.
