# Supplementary material for: Spatiotemporal prediction of wildfire size extremes with Bayesian finite sample maxima
Source: Ecol Appl. 2019 Jun 20;29(6):e01898. doi: 10.1002/eap.1898 (PMC6851762; doi:10.1002/eap.1898)
Supplement: Supplementary file 2 [file EAP-29-na-s002.pdf]

Spatiotemporal prediction of wildfire size extremes with Bayesian finite sample maxima

Appendix S2: Joint distributions

Maxwell B. Joseph*[1], Matthew W. Rossi[1], Nathan P. Mietkiewicz[1], Adam L. Mahood[1], Megan E. Cattau[1], Lise Ann St. Denis[1], R. Chelsea Nagy[1], Virginia Iglesias[1], John T. Abatzoglou[2], and Jennifer K. Balch[1]

[1] *Earth Lab, University of Colorado Boulder*

[2] *Department of Geography, University of Idaho*

Here we provide the unnormalized posterior densities for each model. Square brackets represent a probability mass or density function. Parameterizations for model likelihoods are provided first, followed by the factorization of the joint distribution, with explicit priors.

Poisson wildfire count model

We used the following parameterization of the Poisson distribution:

$$[n|\mu] = \frac{\mu^n e^{-\mu}}{n!},$$

where μ is the mean and variance.

The unnormalized posterior density of this model is:

*maxwell.b.joseph@colorado.edu

$$
\begin{aligned}
&[\boldsymbol{\beta}^{(\mu)}, \alpha^{(\mu)}, \boldsymbol{\phi}, \sigma^{(\phi)}, \eta, \boldsymbol{\lambda}, c, \tau \mid \mathbf{N}] \propto \\
&\prod_{s=1}^{S}\prod_{t=1}^{T}[n_{s,t}|\boldsymbol{\beta}^{(\mu)}, \alpha^{(\mu)}, \phi_{s,t}]\times \\
&[\boldsymbol{\phi}_1|\sigma^{(\phi)}]\prod_{t=2}^{T}[\boldsymbol{\phi}_t|\boldsymbol{\phi}_{t-1}, \sigma^{(\phi)}, \eta]\times \\
&\prod_{j=1}^{p}[\beta_j^{(\mu)}|\lambda_j, c, \tau][\lambda_j]\times \\
&[\sigma^{(\phi)}][\eta][c][\tau][\alpha^{(\mu)}]
\end{aligned}
$$

$$
\begin{aligned}
&= \prod_{s=1}^{S}\prod_{t=1}^{T}\text{Poisson}(n_{s,t}|\exp(\alpha^{(\mu)} + \mathbf{X}_{(s,t)}\boldsymbol{\beta}^{(\mu)} + \phi_{s,t}))\times \\
&\text{Normal}(\boldsymbol{\phi}_1|\mathbf{0}, ((\sigma^{(\phi)})^{-2}(\mathbf{D}-\mathbf{W}))^{-1})\times \\
&\prod_{t=2}^{T}\text{Normal}(\boldsymbol{\phi}_t|\eta\boldsymbol{\phi}_{t-1}, ((\sigma^{(\phi)})^{-2}(\mathbf{D}-\mathbf{W}))^{-1})\times \\
&\prod_{j=1}^{p}\text{Normal}\left(\beta_j^{(\mu)}|0, \frac{\tau^2 c^2\lambda_j^2}{c^2+\tau^2\lambda_j^2}\right)\times \text{Cauchy}^+(\lambda_j|0,1)\times \\
&\text{Normal}^+(\sigma^{(\phi)}|0,1^2)\times \text{Beta}(\eta|1,1)\times \text{Inv-Gamma}(c^2|2.5,10)\times \\
&\text{Normal}^+(\tau|0,5^2)\times \text{Normal}(\alpha^{(\mu)}|0,5^2).
\end{aligned}
$$

Negative binomial wildfire count model

We used the following parameterization of the negative binomial distribution:

$$[n|\mu, \delta] = \binom{n + \delta - 1}{n} \Big(\frac{\mu}{\mu + \delta}\Big)^n \Big(\frac{\delta}{\mu + \delta}\Big)^\delta,$$

where μ is the mean, and δ is a dispersion parameter.

The unnormalized posterior density of this model is:

$$\begin{aligned}
[\boldsymbol{\beta}^{(\mu)}, \alpha^{(\mu)}, \boldsymbol{\phi}, \sigma^{(\phi)}, \eta, \boldsymbol{\lambda}, c, \tau, \delta \mid \mathbf{N}] \propto \\
\prod_{s=1}^{S} \prod_{t=1}^{T} [n_{s,t} | \boldsymbol{\beta}^{(\mu)}, \alpha^{(\mu)}, \phi_{s,t}, \delta] \times \\
[\boldsymbol{\phi}_1 | \sigma^{(\phi)}] \prod_{t=2}^{T} [\boldsymbol{\phi}_t | \boldsymbol{\phi}_{t-1}, \sigma^{(\phi)}, \eta] \times \\
\prod_{j=1}^{p} [\beta_j^{(\mu)} | \lambda_j, c, \tau][\lambda_j] \times \\
[\sigma^{(\phi)}][\eta][c][\tau][\alpha^{(\mu)}][\delta]
\end{aligned}$$

$$\begin{aligned}
= \prod_{s=1}^{S} \prod_{t=1}^{T} \text{Negative Binomial}(n_{s,t} | \exp(\alpha^{(\mu)} + \mathbf{X}_{(s,t)} \boldsymbol{\beta}^{(\mu)} + \phi_{s,t}), \delta) \times \\
\text{Normal}(\boldsymbol{\phi}_1 | \mathbf{0}, ((\sigma^{(\phi)})^{-2} (\mathbf{D} - \mathbf{W}))^{-1}) \times \\
\prod_{t=2}^{T} \text{Normal}(\boldsymbol{\phi}_t | \eta \boldsymbol{\phi}_{t-1}, ((\sigma^{(\phi)})^{-2} (\mathbf{D} - \mathbf{W}))^{-1}) \times \\
\prod_{j=1}^{p} \text{Normal}\left(\beta_j^{(\mu)} | 0, \frac{\tau^2 c^2 \lambda_j^2}{c^2 + \tau^2 \lambda_j^2}\right) \times \text{Cauchy}^+(\lambda_j | 0, 1) \times \\
\text{Normal}^+(\sigma^{(\phi)} | 0, 1^2) \times \text{Beta}(\eta | 1, 1) \times \text{Inv-Gamma}(c^2 | 2.5, 10) \times \\
\text{Normal}^+(\tau | 0, 5^2) \times \text{Normal}(\alpha^{(\mu)} | 0, 5^2) \times \text{Normal}^+(\delta | 0, 5^2).
\end{aligned}$$

Zero-inflated Poisson wildfire count model

We used the following parameterization of the zero-inflated Poisson distribution:

$$[n|\mu, \pi] = I_{n=0}(1 - \pi + \pi e^{-\mu}) + I_{n>0}\pi \frac{\mu^n e^{-\mu}}{n!},$$

where μ is the Poisson mean, and $1 - \pi$ is the probability of an extra zero.

The unnormalized posterior density of this model is:

$$\begin{aligned}
[\boldsymbol{\beta}^{(\mu)}, \alpha^{(\mu)}, \boldsymbol{\beta}^{(\pi)}, \alpha^{(\pi)}, \boldsymbol{\phi}^{(\mu)}, \sigma^{(\phi,\mu)}, \eta^{(\mu)}, \boldsymbol{\phi}^{(\pi)}, \sigma^{(\phi,\pi)}, \eta^{(\pi)}, \boldsymbol{\lambda}, c, \tau, \rho \mid \mathbf{N}] \propto \\
\prod_{s=1}^{S} \prod_{t=1}^{T} [n_{s,t} | \boldsymbol{\beta}^{(\mu)}, \alpha^{(\mu)}, \boldsymbol{\beta}^{(\pi)}, \alpha^{(\pi)}, \phi_{s,t}^{(\mu)}, \phi_{s,t}^{(\pi)}] \times \\
[\boldsymbol{\phi}_1^{(\mu)} | \sigma^{(\phi,\mu)}] \prod_{t=2}^{T} [\boldsymbol{\phi}_t^{(\mu)} | \boldsymbol{\phi}_{t-1}^{(\mu)}, \sigma^{(\phi,\mu)}, \eta^{(\mu)}] \times \\
[\boldsymbol{\phi}_1^{(\pi)} | \sigma^{(\phi,\pi)}] \prod_{t=2}^{T} [\boldsymbol{\phi}_t^{(\pi)} | \boldsymbol{\phi}_{t-1}^{(\pi)}, \sigma^{(\phi,\pi)}, \eta^{(\pi)}] \times \\
\prod_{j=1}^{p} [\beta_j^{(\mu)}, \beta_j^{(\pi)} | \lambda_j, c, \tau, \rho][\lambda_j] \times \\
[\sigma^{(\phi,\mu)}][\sigma^{(\phi,\pi)}][\eta^{(\mu)}][\eta^{(\pi)}][\alpha^{(\mu)}][\alpha^{(\pi)}][\rho] \prod_{m=1}^{2} [c_m][\tau_m]
\end{aligned}$$

$$
\begin{aligned}
= \prod_{s=1}^{S}\prod_{t=1}^{T} \mathrm{ZIP}(n_{s,t}|e^{\alpha^{(\mu)}+\mathbf{X}_{(s,t)}\boldsymbol{\beta}^{(\mu)}+\phi_{s,t}^{(\mu)}}, \mathrm{logit}^{-1}(\alpha^{(\pi)}+\mathbf{X}_{(s,t)}\boldsymbol{\beta}^{(\pi)}+\phi_{s,t}^{(\pi)}))\times \\
\mathrm{Normal}(\boldsymbol{\phi}_1^{(\mu)}|\mathbf{0},((\sigma^{(\phi,\mu)})^{-2}(\mathbf{D}-\mathbf{W}))^{-1})\times \\
\prod_{t=2}^{T}\mathrm{Normal}(\boldsymbol{\phi}_t^{(\mu)}|\eta^{(\mu)}\boldsymbol{\phi}_{t-1}^{(\mu)},((\sigma^{(\phi,\mu)})^{-2}(\mathbf{D}-\mathbf{W}))^{-1})\times \\
\mathrm{Normal}(\boldsymbol{\phi}_1^{(\pi)}|\mathbf{0},((\sigma^{(\phi,\pi)})^{-2}(\mathbf{D}-\mathbf{W}))^{-1})\times \\
\prod_{t=2}^{T}\mathrm{Normal}(\boldsymbol{\phi}_t^{(\pi)}|\eta^{(\pi)}\boldsymbol{\phi}_{t-1}^{(\pi)},((\sigma^{(\phi,\pi)})^{-2}(\mathbf{D}-\mathbf{W}))^{-1})\times \\
\prod_{j=1}^{p}\mathrm{N}\left(\begin{pmatrix}\beta_j^{(\mu)}\\ \beta_j^{(\pi)}\end{pmatrix}\middle|\,\mathbf{0},\begin{pmatrix}\tau_1^2\frac{c_1^2\lambda_j^2}{c_1^2+\tau_1^2\lambda_j^2} & \rho\tau_1\tau_2\sqrt{\frac{c_1^2\lambda_j^2}{c_1^2+\tau_1^2\lambda_j^2}}\sqrt{\frac{c_2^2\lambda_j^2}{c_2^2+\tau_2^2\lambda_j^2}}\\ \rho\tau_1\tau_2\sqrt{\frac{c_1^2\lambda_j^2}{c_1^2+\tau_1^2\lambda_j^2}}\sqrt{\frac{c_2^2\lambda_j^2}{c_2^2+\tau_2^2\lambda_j^2}} & \tau_2^2\frac{c_2^2\lambda_j^2}{c_2^2+\tau_2^2\lambda_j^2}\end{pmatrix}\right)\times \\
\prod_{j=1}^{p}\mathrm{Cauchy}^{+}(\lambda_j|0,1)\times \\
\mathrm{Normal}^{+}(\sigma^{(\phi,\mu)}|0,1^2)\times\mathrm{Normal}^{+}(\sigma^{(\phi,\pi)}|0,1^2)\times \\
\mathrm{Beta}(\eta^{(\mu)}|1,1)\times\mathrm{Beta}(\eta^{(\pi)}|1,1)\times \\
\mathrm{Normal}(\alpha^{(\mu)}|0,5^2)\times\mathrm{Normal}(\alpha^{(\pi)}|0,5^2)\times\mathrm{LKJ}(\rho|3)\times \\
\prod_{m=1}^{2}\text{Inv-Gamma}(c_m^2|2.5,10)\times\mathrm{Normal}^{+}(\tau_m|0,5^2).
\end{aligned}
$$

Zero-inflated negative binomial wildfire count model

We used the following parameterization of the zero-inflated negative binomial distribution:

$$[n|\mu, \delta, \pi] = I_{n=0}(1 - \pi + \pi\Big(\frac{\delta}{\mu + \delta}\Big)^{\delta}) + I_{n>0}\binom{n + \delta - 1}{n}\Big(\frac{\mu}{\mu + \delta}\Big)^{n}\Big(\frac{\delta}{\mu + \delta}\Big)^{\delta},$$

where μ is the negative binomial mean, δ is the negative binomial dispersion, and , and $1 - \pi$ is the probability of an extra zero.

The unnormalized posterior density of this model is:

$$\begin{aligned}
[\boldsymbol{\beta}^{(\mu)}, \alpha^{(\mu)}, \boldsymbol{\beta}^{(\pi)}, \alpha^{(\pi)}, \boldsymbol{\phi}^{(\mu)}, \sigma^{(\phi,\mu)}, \eta^{(\mu)}, \boldsymbol{\phi}^{(\pi)}, \sigma^{(\phi,\pi)}, \eta^{(\pi)}, \boldsymbol{\lambda}, c, \tau, \rho, \delta \mid \mathbf{N}] \propto \\
\prod_{s=1}^{S}\prod_{t=1}^{T}[n_{s,t}|\boldsymbol{\beta}^{(\mu)}, \alpha^{(\mu)}, \boldsymbol{\beta}^{(\pi)}, \alpha^{(\pi)}, \phi_{s,t}^{(\mu)}, \phi_{s,t}^{(\pi)}, \delta]\times \\
[\boldsymbol{\phi}_1^{(\mu)}|\sigma^{(\phi,\mu)}]\prod_{t=2}^{T}[\boldsymbol{\phi}_t^{(\mu)}|\boldsymbol{\phi}_{t-1}^{(\mu)}, \sigma^{(\phi,\mu)}, \eta^{(\mu)}]\times \\
[\boldsymbol{\phi}_1^{(\pi)}|\sigma^{(\phi,\pi)}]\prod_{t=2}^{T}[\boldsymbol{\phi}_t^{(\pi)}|\boldsymbol{\phi}_{t-1}^{(\pi)}, \sigma^{(\phi,\pi)}, \eta^{(\pi)}]\times \\
\prod_{j=1}^{p}[\beta_j^{(\mu)}, \beta_j^{(\pi)}|\lambda_j, c, \tau, \rho][\lambda_j]\times \\
[\sigma^{(\phi,\mu)}][\sigma^{(\phi,\pi)}][\eta^{(\mu)}][\eta^{(\pi)}][\alpha^{(\mu)}][\alpha^{(\pi)}][\rho][\delta]\prod_{m=1}^{2}[c_m][\tau_m].
\end{aligned}$$

$$
\begin{aligned}
&= \prod_{s=1}^{S}\prod_{t=1}^{T} \text{ZINB}(n_{s,t}|e^{\alpha^{(\mu)}+\mathbf{X}_{(s,t)}\boldsymbol{\beta}^{(\mu)}+\phi_{s,t}^{(\mu)}}, \delta, \text{logit}^{-1}(\alpha^{(\pi)} + \mathbf{X}_{(s,t)}\boldsymbol{\beta}^{(\pi)} + \phi_{s,t}^{(\pi)}))\times \\
&\text{Normal}(\boldsymbol{\phi}_1^{(\mu)}|\mathbf{0}, ((\sigma^{(\phi,\mu)})^{-2}(\mathbf{D}-\mathbf{W}))^{-1})\times \\
&\prod_{t=2}^{T}\text{Normal}(\boldsymbol{\phi}_t^{(\mu)}|\eta^{(\mu)}\boldsymbol{\phi}_{t-1}^{(\mu)}, ((\sigma^{(\phi,\mu)})^{-2}(\mathbf{D}-\mathbf{W}))^{-1})\times \\
&\text{Normal}(\boldsymbol{\phi}_1^{(\pi)}|\mathbf{0}, ((\sigma^{(\phi,\pi)})^{-2}(\mathbf{D}-\mathbf{W}))^{-1})\times \\
&\prod_{t=2}^{T}\text{Normal}(\boldsymbol{\phi}_t^{(\pi)}|\eta^{(\pi)}\boldsymbol{\phi}_{t-1}^{(\pi)}, ((\sigma^{(\phi,\pi)})^{-2}(\mathbf{D}-\mathbf{W}))^{-1})\times \\
&\prod_{j=1}^{p}\text{N}\left(\begin{pmatrix}\beta_j^{(\mu)}\\ \beta_j^{(\pi)}\end{pmatrix}\middle|\mathbf{0}, \begin{pmatrix}\tau_1^2\frac{c_1^2\lambda_j^2}{c_1^2+\tau_1^2\lambda_j^2} & \rho\tau_1\tau_2\sqrt{\frac{c_1^2\lambda_j^2}{c_1^2+\tau_1^2\lambda_j^2}}\sqrt{\frac{c_2^2\lambda_j^2}{c_2^2+\tau_2^2\lambda_j^2}}\\ \rho\tau_1\tau_2\sqrt{\frac{c_1^2\lambda_j^2}{c_1^2+\tau_1^2\lambda_j^2}}\sqrt{\frac{c_2^2\lambda_j^2}{c_2^2+\tau_2^2\lambda_j^2}} & \tau_2^2\frac{c_2^2\lambda_j^2}{c_2^2+\tau_2^2\lambda_j^2}\end{pmatrix}\right)\times \\
&\prod_{j=1}^{p}\text{Cauchy}^+(\lambda_j|0,1)\times \\
&\text{Normal}^+(\sigma^{(\phi,\mu)}|0,1^2)\times\text{Normal}^+(\sigma^{(\phi,\pi)}|0,1^2)\times \\
&\text{Beta}(\eta^{(\mu)}|1,1)\times\text{Beta}(\eta^{(\pi)}|1,1)\times \\
&\text{Normal}(\alpha^{(\mu)}|0,5^2)\times\text{Normal}(\alpha^{(\pi)}|0,5^2)\times\text{LKJ}(\rho|3)\times\text{Normal}^+(\delta|0,5^2)\times \\
&\prod_{m=1}^{2}\text{Inv-Gamma}(c_m^2|2.5,10)\times\text{Normal}^+(\tau_m|0,5^2).
\end{aligned}
$$

Generalized Pareto/Lomax burned area model

We used the following parameterization of the GPD/Lomax distribution:

$$[y|\sigma,\kappa] = \frac{1}{\sigma}\Big(\frac{\kappa y}{\sigma}+1\Big)^{-(\kappa+1)\kappa^{-1}},$$

where κ is a shape parameter and σ is a scale parameter.

The unnormalized posterior density of this model is:

$$\begin{aligned}
[\boldsymbol{\beta}, \alpha, \boldsymbol{\phi}, \sigma^{(\phi)}, \eta, \kappa^{(L)}, \boldsymbol{\lambda}, c, \tau \mid \boldsymbol{y}] \propto \\
\prod_{i=1}^{n_{\text{tot}}} [y_i|\boldsymbol{\beta}, \alpha, \phi_{s_i,t_i}, \kappa^{(L)}] \times \\
[\boldsymbol{\phi}_1|\sigma^{(\phi)}] \prod_{t=2}^{T} [\boldsymbol{\phi}_t|\boldsymbol{\phi}_{t-1}, \sigma^{(\phi)}, \eta] \times \\
\prod_{j=1}^{p} [\beta_j|\lambda_j, c, \tau][\lambda_j] \times \\
[\alpha][c][\tau][\kappa^{(L)}][\eta][\sigma^{(\phi)}]
\end{aligned}$$

$$\begin{aligned}
= \prod_{i=1}^{n_{\text{tot}}} \text{Lomax}(y_i|\kappa^{(L)}, e^{\alpha+\mathbf{X}_{(s_i,t_i)}\boldsymbol{\beta}+\phi_{s_i,t_i}}) \times \\
\text{Normal}(\boldsymbol{\phi}_1|\mathbf{0}, ((\sigma^{(\phi)})^{-2}(\mathbf{D}-\mathbf{W}))^{-1}) \times \\
\prod_{t=2}^{T} \text{Normal}(\boldsymbol{\phi}_t|\eta\boldsymbol{\phi}_{t-1}, ((\sigma^{(\phi)})^{-2}(\mathbf{D}-\mathbf{W}))^{-1}) \times \\
\prod_{j=1}^{p} \text{Normal}\Big(\beta_j|0, \frac{\tau^2 c^2 \lambda_j^2}{c^2+\tau^2\lambda_j^2}\Big) \times \text{Cauchy}^{+}(\lambda_j|0,1) \times \\
\text{Normal}(\alpha|0,5^2) \times \text{Inv-Gamma}(c^2|2.5,10) \times \text{Normal}^{+}(\tau|0,5^2) \\
\text{Normal}^{+}(\kappa^{(L)}|0,5^2) \times \text{Beta}(\eta|1,1) \times \text{Normal}^{+}(\sigma^{(\phi)}|0,1^2).
\end{aligned}$$

Tapered Pareto burned area model

We used the following parameterization of the tapered Pareto distribution:

$$[y|\kappa, \nu] = \Big(\frac{\kappa}{y} + \frac{1}{\nu}\Big)\exp(-x/\nu),$$

where κ is a shape parameter and ν a taper parameter.

The unnormalized posterior density of this model is:

$$\begin{aligned}
[\boldsymbol{\beta}, \alpha, \boldsymbol{\phi}, \sigma^{(\phi)}, \eta, \nu, \boldsymbol{\lambda}, c, \tau \mid \boldsymbol{y}] \propto \\
\prod_{i=1}^{n_{\text{tot}}} [y_i|\boldsymbol{\beta}, \alpha, \phi_{s_i,t_i}, \nu] \times \\
[\boldsymbol{\phi}_1|\sigma^{(\phi)}] \prod_{t=2}^{T} [\boldsymbol{\phi}_t|\boldsymbol{\phi}_{t-1}, \sigma^{(\phi)}, \eta] \times \\
\prod_{j=1}^{p} [\beta_j|\lambda_j, c, \tau][\lambda_j] \times \\
[\alpha][c][\tau][\nu][\eta][\sigma^{(\phi)}]
\end{aligned}$$

$$\begin{aligned}
= \prod_{i=1}^{n_{\text{tot}}} \text{Tapered Pareto}(y_i|e^{\alpha + \mathbf{X}_{(s_i,t_i)}\boldsymbol{\beta} + \phi_{s_i,t_i}}, \nu) \times \\
\text{Normal}(\boldsymbol{\phi}_1|\mathbf{0}, ((\sigma^{(\phi)})^{-2}(\mathbf{D} - \mathbf{W}))^{-1}) \times \\
\prod_{t=2}^{T} \text{Normal}(\boldsymbol{\phi}_t|\eta\boldsymbol{\phi}_{t-1}, ((\sigma^{(\phi)})^{-2}(\mathbf{D} - \mathbf{W}))^{-1}) \times \\
\prod_{j=1}^{p} \text{Normal}\Bigg(\beta_j|0, \frac{\tau^2 c^2 \lambda_j^2}{c^2 + \tau^2\lambda_j^2}\Bigg) \times \text{Cauchy}^+(\lambda_j|0, 1) \times \\
\text{Normal}(\alpha|0, 5^2) \times \text{Inv-Gamma}(c^2|2.5, 10) \times \text{Normal}^+(\tau|0, 5^2) \times \\
\text{Cauchy}^+(\nu|0, 1) \times \text{Beta}(\eta|1, 1) \times \text{Normal}^+(\sigma^{(\phi)}|0, 1^2).
\end{aligned}$$

Lognormal burned area model

We used the following parameterization of the lognormal distribution:

$$[y|\mu, \sigma] = \frac{1}{y}\frac{1}{\sigma\sqrt{2\pi}}\exp\Big(-\frac{(\log(y)-\mu)^2}{2\sigma^2}\Big),$$

where μ and σ are location and scale parameters, respectively.

The unnormalized posterior density of this model is:

$$\begin{aligned}
&[\boldsymbol{\beta}, \alpha, \boldsymbol{\phi}, \sigma^{(\phi)}, \eta, \sigma, \boldsymbol{\lambda}, c, \tau \mid \boldsymbol{y}] \propto \\
&\prod_{i=1}^{n_{\text{tot}}} [y_i|\beta, \alpha, \phi_{s_i,t_i}, \sigma] \times \\
&[\boldsymbol{\phi}_1|\sigma^{(\phi)}] \prod_{t=2}^{T} [\boldsymbol{\phi}_t|\boldsymbol{\phi}_{t-1}, \sigma^{(\phi)}, \eta] \times \\
&\prod_{j=1}^{p} [\beta_j|\lambda_j, c, \tau][\lambda_j] \times \\
&[\alpha][c][\tau][\sigma][\eta][\sigma^{(\phi)}]
\end{aligned}$$

$$\begin{aligned}
&= \prod_{i=1}^{n_{\text{tot}}} \text{Lognormal}(y_i|\alpha + \mathbf{X}_{(s_i,t_i)}\boldsymbol{\beta} + \phi_{s_i,t_i}, \sigma) \times \\
&\text{Normal}(\boldsymbol{\phi}_1|\mathbf{0}, ((\sigma^{(\phi)})^{-2}(\mathbf{D}-\mathbf{W}))^{-1}) \times \\
&\prod_{t=2}^{T} \text{Normal}(\boldsymbol{\phi}_t|\eta\boldsymbol{\phi}_{t-1}, ((\sigma^{(\phi)})^{-2}(\mathbf{D}-\mathbf{W}))^{-1}) \times \\
&\prod_{j=1}^{p} \text{Normal}\Big(\beta_j|0, \frac{\tau^2 c^2 \lambda_j^2}{c^2 + \tau^2\lambda_j^2}\Big) \times \text{Cauchy}^+(\lambda_j|0,1) \times \\
&\text{Normal}(\alpha|0, 5^2) \times \text{Inv-Gamma}(c^2|2.5, 10) \times \text{Normal}^+(\tau|0, 5^2) \times \\
&\text{Normal}^+(\sigma|0, 5^2) \times \text{Beta}(\eta|1,1) \times \text{Normal}^+(\sigma^{(\phi)}|0, 1^2).
\end{aligned}$$

Gamma burned area model

We used the following parameterization of the gamma distribution:

$$[y|\kappa, \sigma] = \frac{1}{\Gamma(\kappa)\sigma^{\kappa}} y^{\kappa-1} \text{exp}(-y/\sigma),$$

where κ is a shape parameter and σ a scale parameter.

The unnormalized posterior density of this model is:

$$\begin{aligned}
[\boldsymbol{\beta}, \alpha, \boldsymbol{\phi}, \sigma^{(\phi)}, \eta, \kappa, \boldsymbol{\lambda}, c, \tau \mid \boldsymbol{y}] \propto \\
\prod_{i=1}^{n_{\text{tot}}} [y_i | \beta, \alpha, \phi_{s_i,t_i}, \kappa] \times \\
[\boldsymbol{\phi}_1 | \sigma^{(\phi)}] \prod_{t=2}^{T} [\boldsymbol{\phi}_t | \boldsymbol{\phi}_{t-1}, \sigma^{(\phi)}, \eta] \times \\
\prod_{j=1}^{p} [\beta_j | \lambda_j, c, \tau][\lambda_j] \times \\
[\alpha][c][\tau][\kappa][\eta][\sigma^{(\phi)}]
\end{aligned}$$

$$\begin{aligned}
= \prod_{i=1}^{n_{\text{tot}}} \text{Gamma}(y_i | \kappa, \kappa / \text{exp}(\alpha + \mathbf{X}_{(s_i,t_i)} \boldsymbol{\beta} + \phi_{s_i,t_i})) \times \\
\text{Normal}(\boldsymbol{\phi}_1 | \mathbf{0}, ((\sigma^{(\phi)})^{-2} (\mathbf{D} - \mathbf{W}))^{-1}) \times \\
\prod_{t=2}^{T} \text{Normal}(\boldsymbol{\phi}_t | \eta \boldsymbol{\phi}_{t-1}, ((\sigma^{(\phi)})^{-2} (\mathbf{D} - \mathbf{W}))^{-1}) \times \\
\prod_{j=1}^{p} \text{Normal}\left(\beta_j | 0, \frac{\tau^2 c^2 \lambda_j^2}{c^2 + \tau^2 \lambda_j^2}\right) \times \text{Cauchy}^{+}(\lambda_j | 0, 1) \times \\
\text{Normal}(\alpha | 0, 5^2) \times \text{Inv-Gamma}(c^2 | 2.5, 10) \times \text{Normal}^{+}(\tau | 0, 5^2) \times \\
\text{Normal}^{+}(\kappa | 0, 5^2) \times \text{Beta}(\eta | 1, 1) \times \text{Normal}^{+}(\sigma^{(\phi)} | 0, 1^2).
\end{aligned}$$

Weibull burned area model

We used the following parameterization of the Weibull distribution:

$$[y|\kappa, \sigma] = \frac{\kappa}{\sigma}\Big(\frac{y}{\sigma}\Big)^{\kappa-1}\exp\Big(-\Big(\frac{y}{\sigma}\Big)^{\alpha}\Big),$$

where κ is a shape parameter and σ is a scale parameter.

The unnormalized posterior density of this model is:

$$\begin{aligned}
&[\boldsymbol{\beta}, \alpha, \boldsymbol{\phi}, \sigma^{(\phi)}, \eta, \kappa, \lambda, c, \tau \mid \boldsymbol{y}] \propto \\
&\prod_{i=1}^{n_{\text{tot}}}[y_i|\beta, \alpha, \phi_{s_i,t_i}, \kappa]\times \\
&[\boldsymbol{\phi}_1|\sigma^{(\phi)}]\prod_{t=2}^{T}[\boldsymbol{\phi}_t|\boldsymbol{\phi}_{t-1}, \sigma^{(\phi)}, \eta]\times \\
&\prod_{j=1}^{p}[\beta_j|\lambda_j, c, \tau][\lambda_j]\times \\
&[\alpha][c][\tau][\kappa][\eta][\sigma^{(\phi)}]
\end{aligned}$$

$$\begin{aligned}
&= \prod_{i=1}^{n_{\text{tot}}}\text{Weibull}(y_i|\kappa, \exp(\alpha + \mathbf{X}_{(s_i,t_i)}\boldsymbol{\beta} + \phi_{s_i,t_i}))\times \\
&\text{Normal}(\boldsymbol{\phi}_1|\mathbf{0}, ((\sigma^{(\phi)})^{-2}(\mathbf{D}-\mathbf{W}))^{-1})\times \\
&\prod_{t=2}^{T}\text{Normal}(\boldsymbol{\phi}_t|\eta\boldsymbol{\phi}_{t-1}, ((\sigma^{(\phi)})^{-2}(\mathbf{D}-\mathbf{W}))^{-1})\times \\
&\prod_{j=1}^{p}\text{Normal}\Bigg(\beta_j|0, \frac{\tau^2c^2\lambda_j^2}{c^2+\tau^2\lambda_j^2}\Bigg)\times\text{Cauchy}^+(\lambda_j|0,1)\times \\
&\text{Normal}(\alpha|0,5^2)\times\text{Inv-Gamma}(c^2|2.5,10)\times\text{Normal}^+(\tau|0,5^2)\times \\
&\text{Normal}^+(\kappa|0,5^2)\times\text{Beta}(\eta|1,1)\times\text{Normal}^+(\sigma^{(\phi)}|0,1^2).
\end{aligned}$$
